# In this issue: Volume 113, Issue 5, May 2022

**DOI:** 10.1111/cas.14952

**Published:** 2022-05-24

## Serial circulating tumor DNA monitoring of CDK4/6 inhibitors response in metastatic breast cancer


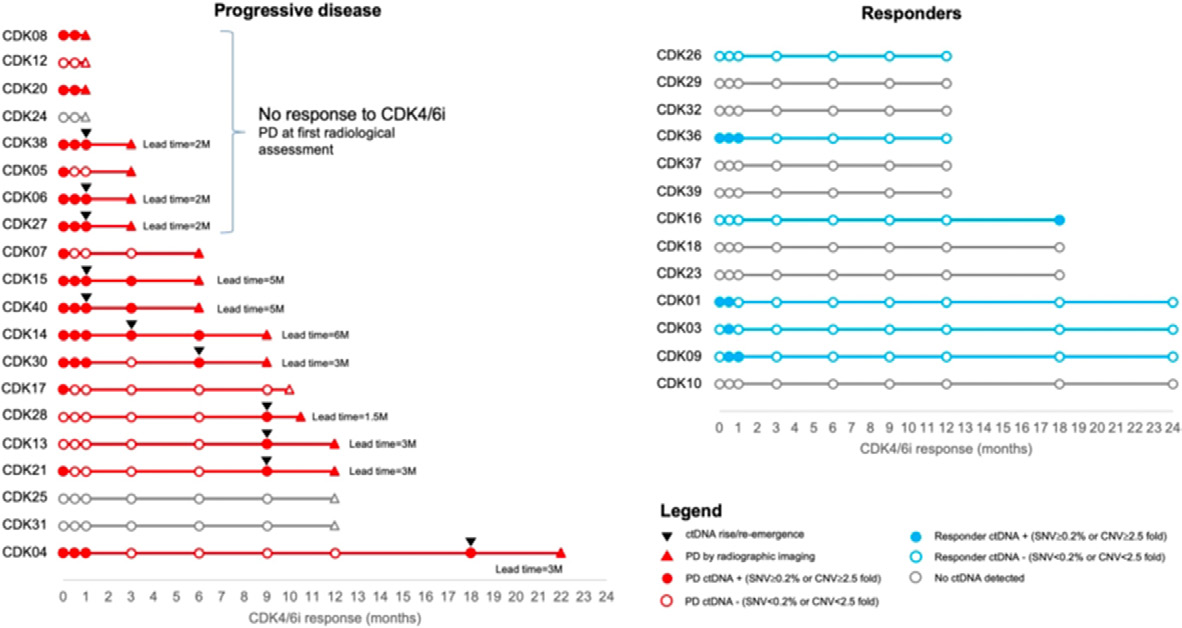


Patients with advanced breast cancer demonstrate significantly improved clinical benefit from treatment with cyclin‐dependent kinase 4/6 inhibitors (CDK4/6i), with some patients developing resistance over time. Treatment response is monitored via radiographic imaging, sometimes with inconclusive findings. Repeated biopsy of the breast cancer tissue is not feasible due to multiple tumor sites and the risky nature of the procedure. A more sensitive and minimally invasive approach is necessary to accurately assess treatment response.

Now, this is possible with the use of circulating tumor DNA (ctDNA). When released into the bloodstream, ctDNA acts as a surrogate marker for cancer cells, providing a ‘real‐time’ snapshot of the disease burden. By measuring ctDNA levels in the bloodstream, along with radiographic imaging assessments, this approach provides deeper insights into how cancer cells are reacting to CDK4/6i.

Chin et al. performed serial ctDNA analysis of blood samples obtained from metastatic breast cancer patients receiving CDK4/6i and endocrine therapy from the Cancer Institute Hospital of the Japanese Foundation for Cancer Research in Tokyo. Their serial ctDNA sequencing approach allows them to establish key landmark intervals for effective monitoring of CDK4/6i treatment response. The dynamic changes of alterations detected through serial ctDNA analysis informed clonal changes associated with tumor response to CDK4/6i. They detected disease progression with 75% sensitivity and 92% specificity. It was especially effective at detecting early progression of the disease, with substantial lead times compared to radiographic imaging assessments.

Their findings are highly promising and strongly establish ctDNA as potential biomarker for treatment effectiveness monitoring in advanced breast cancer. In combination with radiographic imaging, it improves the accuracy of treatment monitoring, especially in complex situations, such as when the tumor size is too small, or has metastasized to the bone. It can also help oncologists manage patients and predict patients that will more likely develop resistance to CDK4/6i.

These findings will pave the way for ctDNA analysis to be established as an effective tool for the management of advanced breast cancer, especially in patients receiving CDK4/6i treatment.

https://onlinelibrary.wiley.com/doi/full/10.1111/cas.15304

## Dahuang Fuzi Baijiang decoction restricts progenitor to terminally exhausted T cell differentiation in colorectal cancer


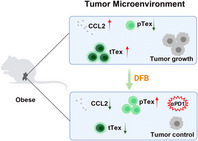


Obesity increases an individual's risk of developing cancer significantly and contributes to almost 40% of cancer‐related deaths worldwide. In particular, the development of colorectal cancer (CRC) is linked directly to obesity. CRC is driven by fat‐storage cells known as adipocytes, which secrete molecules such as chemokines into the surrounding microenvironment. Chemokines in turn enable adipocytes to drive inflammatory responses and compromise the immunity of the microenvironment, thus facilitating tumor development. In response to tumor cell growth, immune cells get alerted, and migrate into the microenvironment. Of these, a subset known as T cells play a key role in recognizing and killing tumor cells.

Unfortunately, prolonged inflammatory responses may cause T cells to get exhausted. These exhausted cells show an increase in the proteins PD‐1(Programmed death receptor‐1) and Tim3 (T‐cell immunoglobulin and mucin domain‐containing protein 3) on their cell surface, and have a diminished capacity to kill tumor cells.

In this study, Xu et al. tried to understand if Dahuang Fuzi Baijiang (DFB), which is a Chinese herbal formula known for its medicinal properties, can prevent the exhaustion of T cells. To test this hypothesis, they assessed the efficacy of DFB in mice which were fed with a high‐fat diet, to mimic the development of obesity in humans. They induced tumors in these mice as well as in mice on a normal diet, and compared tumor growth between them. They found that tumor growth was much higher in high‐fat diet‐fed obese mice than in the control mice. Moreover, in obese mice, treatment with DFB suppressed the tumor growth.

T cells in the tumor microenvironment of obese mice expressed high amounts of PD‐1 and Tim3 (PD‐1hiTim3+), which did not contribute to tumor killing. After being treated with DFB, these cells displayed lower levels of PD‐1. Interestingly, these T cells also expressed another factor known as Tcf1 (PD‐1intTcf1+) on their surface and retained some of their anti‐tumor activity. Further assessments helped the authors understand that DFB decreased the adipocyte‐induced release of chemokines and prevented T cells from entering an exhausted state. What's more, combining DFB with additional treatments, such as anti‐PD‐1 drugs, enhanced the anti‐tumor activity of T cells.

These findings highlight a novel treatment strategy for CRC using a traditional herbal formula, which shows great promise to be established on a large scale, following clinical verification.

https://onlinelibrary.wiley.com/doi/10.1111/cas.15311

## Novel antiangiogenic therapy targeting biglycan using tumor endothelial cell‐specific liposomal siRNA delivery system


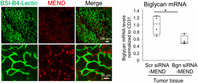


The formation of new blood vessels, or angiogenesis, plays a key role in the growth of tumor cells. Tumors release certain angiogenic factors, which increase the formation of new capillaries required for their growth and sustenance. For this reason, antiangiogenic therapy, which prevents the formation of new blood vessels or capillaries, is effective at suppressing tumor growth.

The main targets of antiangiogenic therapy are tumor endothelial cells (TECs), which line the inner surface of tumor blood vessels. Recently, it was observed that the expression of a signaling molecule known as biglycan was high in the TECs of certain tumors. TECs utilize biglycan to enhance angiogenesis. In turn, biglycan facilitates the movement of tumors through newly formed blood vessels, enabling metastasis. Based on these findings, biglycan shows potential as an important therapeutic target for cancer treatment. However, there are no commercially available inhibitors of biglycan yet.

Through this study, Maishi et al. tried to understand if the inhibition of biglycan in the TECs of renal cell carcinoma had any therapeutic benefit. To do this, they developed a nano drug delivery system (DDS) termed the multifunctional envelope‐type nanodevice (MEND). First, they used a special peptide to deliver MEND containing small interfering RNAs into the TECs of the renal cell carcinoma tissues. They confirmed that TEC‐targeting MEND entered the renal TECs and inhibited the expression of biglycan by silencing the gene that codes for it.

Next, the authors evaluated the efficacy of TEC‐targeting MEND in tumor‐bearing mice. They found that it targeted their TECs as well, leading to a reduction in the expression of biglycan within the cells. In fact, biglycan inhibition in mice not only suppressed angiogenesis, but also normalized the tumor microenvironment by alleviating hypoxia and fibrosis, which usually enhance tumor growth.

Their findings clearly highlight that biglycan inhibition is a novel and effective strategy for cancer treatment and should be explored further.

https://onlinelibrary.wiley.com/doi/full/10.1111/cas.15323

